# Perceived facilitators of and barriers to mental health treatment engagement among decision-making competent adolescents in Greece

**DOI:** 10.1186/s12888-021-03471-0

**Published:** 2021-09-22

**Authors:** E. Tsamadou, P. Voultsos, A. Emmanouilidis, G. Ampatzoglou

**Affiliations:** 1 Department of Child & Adolescent Psychiatry, Hippokration General Hospital of Thessaloniki, Konstantinoupoleos 49, Postal Code 546 42 Thessaloniki, Greece; 2 Laboratory of Forensic Medicine & Toxicology (Medical Law and Ethics), School of Medicine, Faculty of Health Sciences, Aristotle University, University Campus, Postal Code 541 24 Thessaloniki, Greece; 3 Department of Child & Adolescent Psychiatry, School of Medicine, Faculty of Health Sciences, Aristotle University, Campus, Postal Code 541 24 Thessaloniki, Greece

**Keywords:** Adolescent psychotherapy engagement, Shared decision making, Decision-making competent adolescents, (psycho-) therapist

## Abstract

**Background:**

A subset of adolescents with mental disorders are likely to have decision-making capacity that facilitates their therapy engagement. However, there are high rates of drop-out in mental health settings.

**Aim:**

This study aims to identify perceived barriers to or facilitators of mental health care engagement among adolescents with decision-making competence in Greece.

**Methods:**

A qualitative study was conducted using semi-structured interviews of adolescents with a wide range of mental health problems. In addition, two psychometric assessment measures were used to define who to include or exclude from the study sample.

**Results:**

Positive attitudes and experiences with therapy were reported as strong (“major”) facilitators of therapy engagement for adolescents with mental disorders, whereas negative experiences with therapy were reported as strong barriers to it. Furthermore, and most importantly, a “good” adolescent-therapist relationship was reported as a strong facilitator, whereas negative experiences of participants with their therapist were reported as strong barriers. Moreover, goals such as getting rid of symptoms, improving personal well-being, and improving social skills and relationships (especially with peers) emerged as strong facilitators of therapy engagement. Importantly, the early remission of symptoms emerged from the study as a strong barrier to therapy engagement for participants. Among the weaker (“minor”) perceived facilitators were goals such as confessing to a trustworthy person, becoming able to achieve personal expectations and life goals, enhancing independence and self-esteem, and developing a positive self-image. The (active or supportive) role of family emerged as a facilitator. The stigma related to mental health emerged as both a (“minor”) facilitator of and barrier to therapy engagement for participants. Friends were reported as having a role ranging from neutral to mildly supportive.

**Conclusion:**

A number of more or less strong barriers and facilitators were identified that, for the most part, were consistent with prior literature. However, the authors identified some nuances that are of clinical importance. For instance, adolescents are most likely to terminate the treatment prematurely if they experience early symptom remission. Highlighting the role of therapy in achieving their goals or improving their families’ well-being might be used by therapists to reduce the attrition rate.

**Supplementary Information:**

The online version contains supplementary material available at 10.1186/s12888-021-03471-0.

## Background

### Adolescent psychotherapy engagement and decision-making capacity

Adolescence is a unique period of transition between childhood and adulthood, with multidimensional changes that make adolescents susceptible to mental health disorders. According to worldwide community studies, the pooled prevalence of any mental disorder in adolescents is 13.4%, with prevalence rates ranging between 8.3 and 19.9% [1]. In general, patients have trusted, personal relationships with their doctors in patient-focused care models, at the core of which is shared decision making (SDM). Decision-making competent adolescents with mental disorders might be likely to develop good relationships with their therapists, which, when containing a degree of mutuality, is important and can positively influence engagement with therapy. As presented below, this is an important predictor of youth client retention in therapy. Retention in therapy is one of the strongest predictors of improved outcomes among adolescents with mental disorders. Attrition refers to dropping out of (outpatient) mental health therapy early, namely, terminating therapy before the therapist would agree that it is appropriate to do so, thus leading to an attenuation of therapy outcomes.

A high attrition rate is a problem in the field of child and adolescent psychiatry. It is noteworthy that there are high rates of drop-out in mental health settings [2]. “Youth are particularly difficult to engage” [3]. Warnick et al. stated that ‘attrition in youth outpatient mental health clinics ranges from 30 to 70% and often occurs early in treatment’ [4]. The “good” adolescent-psychotherapist relationship involves a positive and reciprocal interaction between adolescents and therapists and is a key component of effective psychotherapy engagement, especially in the field of child and adolescent psychiatry [5]. SDM “is increasingly being suggested as an integral part of mental health provision” [6], especially in the context of child and adolescent psychiatry [7, 8]. The SDM process may contribute to creating optimum collaborative working involvement between the therapist and adolescent, namely, a “good”, interactive and effective adolescent-psychotherapist relationship. An adolescent patient must have decisional capacity to be an active participant in the SDM process. SDM is an interactive process that emphasizes the patient’s values and preferences, in which psychotherapists and patients work together to make decisions in line with the principles of person-centred care [8]. Psychotherapists balance risks and expected outcomes with patient preferences and values. This process epitomizes decision-making involvement, where each patient is given the opportunity to share their opinions with psychotherapists.

### Decision-making capacity in psychiatry

Mental disorder does not necessarily involve a loss of decision-making competence [9]. It is important to bear in mind that perfect cognitive functioning is not necessarily a requirement for decision-making capacity (DMC) [10]. Nevertheless, perfect cognitive functioning is not always sufficient to have full DMC. Modern decision-making theory places considerable emphasis on values and emotions rather than on cognitive function when considering one’s DMC [11]. Having developed a set of core values is a requirement for DMC.

Interestingly, it has been suggested that practical wisdom, namely, “knowing the right thing to do in the concrete situation”, might be a reliable criterion for assessing an individual with mental disorders as decision-making competent. The ability of such patients to organize their core values, find a balance between extreme emotions, and enact their core values and emotions in what they consider a good and meaningful personal life has been proposed as the criterion for having practical wisdom [12]. Note, however, that the criterion of practical wisdom for DMCs has not yet been commonly accepted and remains to be further investigated in the context of DMCs.

### Decision-making capacity in adolescence

Adolescence is a culturally defined concept without clear-cut starting and ending points [13]. Adolescents should be involved in treatment decisions to the extent possible [14, 15]. Their decision-making competence should be assessed on an individual basis. Below, the authors of this paper discuss this topic in detail.

Studies have demonstrated that a subset of adolescents aged 14 and older have the capacity to consent to medical treatments in specific contexts [16, 17]. Adolescents demonstrate noticeable DMC across a variety of domains [18]. Legally binding international texts such as the United Nations Convention on Rights of the Child (articles 12 and 13) and the Convention on Human Rights and Biomedicine (article 6) provide that the voices of children and adolescents should be heard and given due weight. Indeed, the right of adolescents to be involved in treatment decisions has been expanded in recent years [19]. Adolescents’ involvement in medical decisions is important to them. Decision-making involvement helps them become better decision-makers in the future [15] and learn that they are beings of ‘moral worth’ [20].

Regarding adult patients, it is extremely difficult to define a cut-off point of consent for medical treatment [21]. That is particularly so in regard to adolescents. There is no universal agreement on adolescents’ decision-making capacity (DMC). There is no chronological age of consent for medical treatment [22–24]. The existing consent frameworks do not specify a minimum age at which an adolescent might be considered competent to consent to medical treatment [25]. Importantly, it seems impossible to define a cut-off point of consent for medical treatment based on neuroscience [26, 27].

Adolescences’ decision-making competence is determined by a variety of factors related to adolescents themselves (i.e., developmental stage, literacy, culture, experience, health state), their context (i.e., parents that may be facilitators or barriers to adolescents’ decision-making competence, peers, health professionals and the relationships between the adolescent and these individuals), and other situational factors [28, 29]. Importantly, there is uniqueness and diversity in adolescence. Asymmetry in the development of various structures in the adolescent brain is the main factor responsible for making adolescence a unique developmental period requiring a tailored response [27]. Furthermore, other authors highlight the role of factors such as the development of skills and mature critical thinking; the development of values, emotions and moral authority; literacy; culture; previous experiences; the involvement of family; and family relationships [14, 30–35]. Last, as briefly mentioned above, adolescents’ decision-making competence fluctuates. It is argued that ‘decision-making, even of mature adolescents, may occasionally be flawed’ [36]. Furthermore, there are considerable individual differences in rates of developmental maturation among adolescents [5]. Moreover, adolescents are profoundly influenced by other persons, especially parents, health professionals and peers [37, 38].

Adolescents’ medical decision-making process is much more complex than adults’ medical decision-making process, and this is particularly the case in regard to adolescents with mental disorders [19].

### The aim of this study

Greece was ravaged by financial crisis from 2009 to 2016. The recent economic crisis may have profound and lasting effects on the mental health of adolescents. Adolescents’ behavioural health is particularly vulnerable to conditions of economic hardship [39]. Reiss found that “socioeconomically disadvantaged children and adolescents were two to three times more likely to develop mental health problems” [40]. Child and adolescent psychotherapy services may contribute to buffering against these situations. Note, however, that Greek researchers have reported high rates of non-attendance at psychotherapy among adolescents in the country. Kontorini et al. noted that out of 319 children and adolescents (131 girls and 188 boys) who sought psychotherapeutic help in the Department of Children and Adolescents’ Therapy (DCAT) of the Institute of Behaviour Research and Therapy (IBRT) during the last decade (January 2010–May 2020, namely, the decade after the economic crisis), 42.6% successfully completed it, 20.7% did not attend and 13.5% dropped out [41].

To the best of the authors’ knowledge, little research has been conducted to date on factors that may be barriers to or facilitators of mental health care engagement among decision-making competent adolescents in Greece. In the absence of adequate specific empirical knowledge about this topic, the authors sought to identify those factors that adolescents with mental disorders who, in their assessment, showed a reliable level of DMC regard as facilitators of or barriers to engaging in psychotherapy. This study, along with another study entitled “Clinical characteristics of adolescents undergoing psychotherapy” were parts of a broader research project exploring the topic of consensual mental health treatment engagement among adolescents.

### Research questions

The overarching question delineating the focus of this study was the following:

What are the perceived facilitators of and barriers to psychotherapy engagement among adolescents who show a reliable level of decision-making capacity?

The secondary research questions were the following:

- What are the major factors that can profoundly shape the attitude of adolescents with mental disorders towards their treatment?
- What are the factors that determine the consent of adolescents with mental disorders to their treatment?
- What are the factors that determine the treatment decision making of adolescents with mental disorders?

### Study design

The present work was a prospective qualitative research study based on in-depth interviews with adolescent outpatients with psychiatric disorders. This qualitative descriptive study was conducted from June 2016 to December 2019 using a conventional content analysis approach. Moreover, screening activities to determine participant eligibility were performed following their consent to continue with the research while protecting the privacy of potential participants and the confidentiality of information collected about them. The screening activity was divided into two stages.

### Stages of the study

A. A psychometric evaluation of the adolescent patients who were willing to participate in this study was conducted for the primary purpose of applying inclusion and exclusion criteria to the research sample. This was the first stage of participant eligibility screening. The psychometric evaluation was performed after obtaining informed consent (enrolment) to ensure that participants were qualified for the study. In addition to eligibility screening*,* the psychometric assessment was expected to help us identify more factors potentially affecting the patients’ attitude towards their treatment and carry out a more nuanced and reliable qualitative analysis, namely, obtain better results from our qualitative data analysis*.*

B. Clinical assessment was the second stage of participant eligibility screening. It was assessed whether participants had adequately developed emotions and a set of values as well as the abilities to organize their values and find a balance between extreme emotions.

C. After having undergone the psychometric evaluation, the members of the research sample participated in in-depth interviews. The authors conducted in-person interviews based on a semi-structured interview guide. Then, an inductive content analysis was conducted.

### Participants

#### Sampling and data collection

In the present study*,* the authors focused on adolescent patients’ perceptions and attitudes to identify motivating factors for outpatient adolescents that may help them engage with their treatment. Purposive sampling was used according to the eligibility criteria set out below.

The participants were outpatients attending their scheduled clinic appointments in the Child and Adolescents Psychiatry Department, tertiary referral hospital Hippokratio of Thessaloniki. Potential participants were approached by the lead researcher (ET) at the clinic. A broad range of mental disorders were included, such as depression, obsessive-compulsive disorder, dysthymia, behavioural disorders, anorexia nervosa, panic disorder, bipolar disorder, and autism spectrum disorder. All of the participants had been attending psychotherapy for at least four months, and most of them had been in psychotherapy for more than six months. None of them were involuntarily referred by parents or other caregivers.

Thus, participants who differed in age, gender, educational background, experience and type of mental disorder were recruited continuously during the project by the main researcher (ET). The researchers achieved a cluster random sample. To gain a deep and comprehensive understanding of the underlying phenomena that drive psychiatric adolescents’ attitudes towards their own treatment, the researchers strove to construct an overall sample consisting of participants with a wide range of mental health conditions, namely, corresponding to a wide range of types of mental disorder, weighed against a practical wish to recruit the greatest possible number of participants with each type of mental disorder.

A total of 52 patients were initially recruited by the main researcher (ET). While 50 patients agreed to be interviewed, 2 patients chose not to participate. None of the patients initially agreed to participate and then changed their mind and declined participation.

#### The inclusion criteria

The inclusion criteria for participation in the study were (1) being an outpatient with mental disorders, (2) being adolescents less than 18 years, (3) being already engaged in a therapeutic relationship in our healthcare setting, and (4) being not involuntarily engaged in this therapeutic relationship.

#### The exclusion criteria

The exclusion criteria were designed to create a pool of assessed-as-decision-competent participants who were consensually engaged in ongoing treatment for at least four months at the time of the interview.

The exclusion criteria for participation in the study were (1) inability to use the Greek language and (2) having a very low literacy level (determined empirically), namely, a literacy level below what is needed for effective interviewing. Adolescents’ decision-making competence was assessed on an individual basis. To determine whether the participants had adequate capacity to make decisions, the researchers examined their cognitive functioning, emotions, core values, and practical wisdom to the extent possible. Therefore, the following exclusion criteria were further included: (3) the lack of an adequately developed set of values (explored through participant screening interviews conducted prior to the qualitative research interviews, as presented below), (4) very low intelligence or (5) severe depression symptoms that may have a negative impact on cognitive functions and decision-making processes. Therefore, patients with severe disorders in cognitive functioning or severe emotional (major depressive) disorders were excluded. These disorders may not only have a profoundly negative impact on participants’ DMC but also reduce the reliability and effectiveness of interviews.

### Procedures

#### Informed consent

As participants were under 18, written informed consent was obtained from a parent and/or legal guardian. In addition, verbal informed consent was obtained from adolescent participants. Both parents/legal guardians and adolescents were told at the start of the study that they have the right to withdraw from the research at any time and without giving any reason and without reprisal. Before each interview, each participant and his or her parent(s) were given information on the study and informed that his or her participation was voluntary, placing great weight on the importance of maintaining confidentiality.

#### Screening

##### Screening measures

Potential participants were administered two psychometric scales: A) An intelligence test (Wechsler Intelligence Scale for Children, WISC III) [42] and a self-report measure of depression (*Beck Depression Inventory, BDI* II) [43]. The Greek versions of both psychometric tests were considered reliable since these questionnaires have been validated in the Greek context [44, 45]. ET was the researcher who administered the WISC and BDI psychometric scales to potential participants. All the requirements of quantitative and qualitative research were met, and all ethical considerations were observed.

##### The procedures of participant screening

In applying inclusion and exclusion criteria to our sample, the researchers determined a threshold value (cut-off points) to identify those patients with “very low intelligence” or “severe depression symptoms”, namely, those who were not *eligible to become participants* in the qualitative research. The cut-off point used for the WISC III was < 54, and the cut-off point used for the BDI II was > 31. If the total score was less than these threshold values, the intelligence and depression symptoms were ranked as “very low” or “severe”, respectively.

Then, in a second step, adolescents whose intelligence and depression symptoms were not ranked as “very low” or “severe”, respectively, were clinically assessed to determine whether they had developed a set of (not pathogenic) values strictly and stably over time allied to their narrative identity as well as whether they had adequate (though not extreme) emotions. The assessment was not difficult provided that the adolescents had already been in therapy for many months, namely, at least four months, though most had been in therapy six months. Moreover, the researchers determined whether they had developed practical wisdom, namely, whether they were capable of focusing on “knowing the right thing to do in the concrete situation” [12]. More precisely, the researchers clinically assessed whether they had developed a set of core values and whether they were able to organize their core values, find a balance between extreme emotions, and enact their core values and emotions in what they considered a good and meaningful personal life [12]. The narrative enabled the researchers to enter into the being (inner world) of participants to appreciate these abilities. To determine whether these (additional) criteria were met, the researchers conducted participant screening interviews with potential participants, conducted prior to the qualitative interviews albeit after the psychometric assessment. The screening interview guide touched upon many points to capture all of the aforementioned dimensions of practical wisdom. For instance, potential participants were encouraged to talk about their lives and future plans and comment on their value-laden and emotion-provoking assumptions regarding hot social topics, such as racism or homosexuality. This purpose of this process was to induce them to find, reflect on and deal with their core values, express their feelings and manage their emotional reactions. Many participants made their comments using emotion-laden words. Furthermore, nonverbal reactions and interactions were given particular weight and were analysed. Nonverbal reactions were often directly connected to verbalized emotions. In addition, the participant screening interviews were rapport-building. While the participants in this study did not lack the capacity for cognitive reasoning and had adequate values and emotions, the researchers were of the view that having practical insight and being able ‘to handle the situation and live a meaningful life’ might further underpin their judgement about the DMC of the adolescents who were considered eligible to participate in the study. This was because adolescents with mental disorders are in an extremely grey zone between competence and incompetence. The researchers used the ICD-10 to obtain a diagnosis for mental health disorders.

#### Interviews

The interview guide reflected the overarching aim of the study: to investigate any barriers to and facilitators of adolescents’ capacity to decide and give valid consent to their treatment. The interviews were semi-structured and started by guide questions such as the following: What are your views about your treatment? How did you decide whether you should undergo treatment? Please describe to me any significant experience you have had dealing with your problems. Have you ever felt concerned about your treatment? What do you believe are the main reasons why you have to comply with the treatment offered? What is or should be the role of your therapist and the relationship between you and your therapist? The interview guide questions were developed specifically for this study. The questions were tailored to the participants’ condition, with the possibility of qualifying probing questions that were not listed in the interview guide, depending on the course of the interview. The topics set out in the interview guide included reasons, feelings, views, experiences and understanding. The interviews were focused, among other things, on elucidating the reasons why patients decided to undergo medical treatment, their comprehension relating to treatment design and processes, and their experiences of taking part in the therapeutic alliance with their therapist and receiving a drug. Moreover, the interviews included questions about the participants’ experiences and perceptions of treatment, as well as questions about their view of social and family relationships, future targets and goals. Study participants were also asked about difficulties complying with the treatment offered.

As data analysis commenced while the trial was ongoing, new topics were highlighted during the initial few interviews and emerged from the data analysis. In subsequent interviews, these topics became the subject of additional questions, which were added to the interview guide to further confirm the trustworthiness of the research data.

Participants were encouraged to expand upon issues they considered most relevant and speak as freely as possible about them. Participants were encouraged to elaborate on their initial responses to the questions. The interviewer made every effort not to interrupt the interviewer while speaking or to disturb the interviewer while remaining silent. It should be highlighted that the interviewer tried her utmost not to ask leading questions.

All interviews were carried out in person by the first author (ET), a specialist in child and adolescent psychiatry possessing a Master of Medical Ethics and experienced in qualitative interviewing. Data were collected through in-depth individual semi-structured interviews. Relevant field notes were written before and after the interviews by the interviewer to help produce a comprehensive set of insightful findings.

Patients were given the choice of being interviewed either at their home or at their therapist’s general practice. Interviews were held at interviewees’ preferred time. During the interview, only the interviewer and the interviewee were present, with the exception of a few patients who were accompanied in the interview by their parents, who for the most part stayed silent apart from brief input. This input was not analysed and used for the paper.

The interview language was Greek, and interviews were conducted between June 2016 and December 2019. Interviews lasted between 30 and 45 min. The mean length of the interviews was 35 min. Interviews were audio recorded and transcribed verbatim by the first author into written Greek*.*

Although the researcher had a clinical background, she assumed the researcher role for the interviews. The interviewer did not hold any strong views about medical ethics and remained neutral on issues that were discussed with the patient. The researcher contacted the patients and spent time with them beforehand to gain their trust and ensure trustworthiness in the study. The interviewer answered any questions that the patients asked*.* Reflexive thinking was applied throughout the research process to reduce unintentional personal bias. The researcher took great care to make the interviews feel more like a conversation and less like an interrogation, especially from the adolescent patients’ perspective.

The number of participants was not set from the beginning. Data collection ceased only when data saturation was reached, a point where no additional information was obtained from further interviews. Data collection continued until after forty-five interviews. Five more interviews were also conducted to ensure data saturation.

#### Data analysis

The analysis was mainly performed by the first author. The co-authors contributed to the analysis from their respective points of view as a bioethicist, clinician, or psychologist. Authors engaged with one another to limit research bias.

As the data did not involve particularly large volumes of text, the researchers placed great weight on the interpretation of the ‘latent content’ of the texts and on the subjective understanding of “patterns, themes, and categories important to asocial reality” [46]. The interviews were analysed with conventional qualitative content analysis, as suggested by Graneheim and Lundman [47]. With respect to the aim of the study, the analysis was focused on identifying the perceptions of adolescent patients regarding their treatment. In this sense, the analysis was purposive.

The researchers took great care to ensure the validity and reliability of the study according to Gibbs [48]. In addition, the researchers used strategies to minimize reflexivity throughout the project. For instance, throughout the content data analysis, the researchers drew upon our own clinical experiences in a reflexive manner. Furthermore, credibility was established using prolonged engagement and maximum variance in participant selection. Moreover, transferability was achieved via the provision of a rich description of the data collection, analysis processes and findings to allow the readers to match the findings with their contexts. The trustworthiness of a qualitative research study involves establishing credibility and transferability as well as dependability, confirmability and authenticity [49, 50].

As the purpose of the study was not early theory testing, the researchers used inductive content analysis, namely, an approach moving from the specific to the general [51].

After each interview was transcribed word for word, in the first step, each interview transcript was read through carefully and repeatedly to obtain a good overall sense of the whole transcript and an impression of its content [52]. In the second step, units expressing meaning were identified in each interview transcript, and units similar in meaning were coded. The researchers constantly compared data to ensure that the codes were used consistently [53]. In the third step, codes similar in meaning were grouped into subcategories. In the fourth step, subcategories compared with each other and the latent data content were condensed into broader categories. The final categories were refined by all authors by ensuring a clear difference between categories and subcategories. The lists of categories were grouped under higher-order headings. The categories were grouped into prevailing themes as the final product of the analysis. Disagreements between the authors that arose during the data analysis were easily addressed with re-examination of the data and further discussion.

Data analysis was carried out using NVivo qualitative data analysis software version 9 released in 2010.

Last, it should be noted that facilitators of and barriers to psychotherapy engagement that emerged from this study were ranked as major (strong) and minor (less strong). Note, however, that there were no clear cut-off criteria for the clarification of major and minor barriers or facilitators. Barriers or facilitators that were highly recurrent or mostly emphasized during the interviews were classified as major factors. Barriers or facilitators that were not highly recurrent or mostly emphasized during the interviews were classified as minor factors. From this perspective, the potential of the researchers (with previous experience in qualitative data analysis) to make accurate judgements about whether a barrier or facilitator can be classified as major or minor plays a crucial role.

### Ethical considerations

The authors obtained adolescent consent and parental consent for adolescent participants. If adolescents and their parents were willing to participate, they were given adequate information about the design, purpose, nature and confidentiality of the study, including that participation was voluntary and that consent could be withdrawn at any time during the course of the study. Verbal informed consent to participate was then obtained from each participant and his or her parent(s) prior to participating in this study and documented in the recording at the time of the interviews. Anonymity and confidentiality were maintained throughout the study. Interview data were anonymized during transcription. To preserve participants’ anonymity, no names are used in this paper. The interviews were registered and stored in a strictly confidential fashion. The study and consent procedure were approved by the Ethics Committee affiliated with Aristotle University of Thessaloniki, Faculty of Health Sciences, School of Medicine (Decision Number: 9.302 / 12-07-2017).

## Results

Qualitative interview data were collected from a purposive sample consisting of 50 (assessed as) decision-making competent adolescents who were in ongoing outpatient psychotherapy*.* A total of 50 adolescents (aged 13–18 years, mean = 14.85, SD = 1.67) with psychiatric disorders participated in the study (see Table 1).

**Table 1:** Mean scores, standard deviations, maximum and minimum values of the age of participants

	M.S.	S.D.	Min.V.	Max.V.	n
Age	14.85	1.67	12.0	18.0	50
Boy	14.97	1.67	13.0	17.5	22
Girl	14.75	1.70	12.0	18.0	28

M.S. = Mean Scores, S.D. = Standard Deviation, Min.V. = Minimum Value, Μax.V. = Maximum Value

From the interview data analysis emerged a variety of distinct factors that can be regarded as barriers to and facilitators of psychotherapy engagement for adolescents with mental disorders who are decision-making competent. Below are the six central themes and the sub-themes that emerged from the interview data analysis [for additional representative quotes, see Additional File 1]. Note that the facilitators of and barriers to psychotherapy engagement that emerged from this study were ranked as major (strong) and minor (less strong) according to their relative recurrence rate in the interviews (quantitative criterion) and the emphasis placed on these interview data by participants (qualitative criterion).

### The adolescents’ attitudes towards therapy

#### Strong commitment to therapy

In the sample of adolescents examined, almost all participants were undoubtedly in favour of therapy. They expressed their strong willingness to undergo it.

The vast majority of participants not only were committed to therapy but also invested in it to varying degrees. One participant, while recognizing the beneficial role of therapy and declaring her commitment to it, stressed that she would avoid investing in therapy.

*“I don’t think of anything special, it’s a new experience as well, which will help me; it will get somewhere, but I don’t need to have a special feeling for it … are you scared of something? fast answer: No” (participant 8, girl 15 years old, F50)*

However, some participants were strongly attached to treatment, had invested in it, and hence found it difficult to see reasons to interrupt it. Typical comments included,

*“Nothing would make me stop treatment” (Participant 31, girl 16yo, F50)*

#### Ineffectiveness of therapy as a barrier

Perceived ineffective treatment may be a good reason for terminating therapy prematurely. The following is one of the comments illustrating this point:

“*… [I’d interrupt the treatment] if it is extremely difficult to follow it or I didn’t notice any result in some time” (Participant 4, girl 17yo, F50)*

Some participants related the perceived ineffectiveness of therapy to their therapist. For example, one participant said,

*“… the relationship with the therapist made me continue … if it didn’t help me [I’d stop treatment]” (Participant 2, boy 13yo, F51.3)*

### The crucial role of the adolescent-therapist relationship

It was strikingly apparent in the data analysis that the adolescent-therapist relationship plays a crucial role in adolescents’ therapy engagement. This relationship may be regarded as both a strong barrier (when it is “bad”) or a strong facilitator (when it is “good”). The adolescent-therapist relationship appears to be very specific and essential for achieving adolescents’ treatment engagement and hence effective treatment. Establishing an adolescent-therapist relationship perceived as ‘good’ by adolescents may be a strong facilitator of adolescents’ treatment engagement, whereas a perceived ‘bad’ relationship with the therapist constitutes a strong barrier to adolescents’ treatment engagement.

In the beginning of psychotherapy, the adolescent is initially cautious and curious, expecting that the therapist will persuade him or her to collaborate. Adolescents initially set the precondition of “comfort” with their therapist so as to “succumb” to therapeutic guidance. Adolescents search for specific characteristics in the therapist. They require that their therapists have a warm and friendly demeanour and strive to create a trustful relationship with their adolescent patients. Trust in the therapist and in the promise of confidentiality are important elements for the continuation of the therapeutic relationship and a prerequisite for adolescents. Personal liking for the therapist is often an essential condition for continuing treatment. “If I didn’t like her as much as I do, I’d be more hesitant to continue treatment”. The need for confidentiality to prevent social comments is a required characteristic of their relationship with the therapist. Adolescents need to feel the specialist is close to them and a sense of feel confidence, comfort and intimacy and that the therapist speaks “the same language” as them so they do not feel alone in their mental illness. However, adolescents require that the therapist does not play the role of an expert dictating the life of the adolescent through a paternalistic model of the doctor-patient relationship. Shared decision making is desirable. It is indicative that two participants used the terms “collaborate [with the therapist]” (participant 33, girl 17yo, F42) and “[the therapist] is cooperative” (participant 20, girl 18yo, panic attacks).

#### The (perceived as) ‘good’ adolescent-therapist relationship

The vast majority of participants in this study did not describe any actual concerns about their therapist. Participants who perceived the relationship with their therapist as ‘good’ stated that their therapist was “very good” and that they had developed a friendly relationship of trust with him or her.

A typical comment was:

*“Our relationship is good, she is friendly, I can trust her with many things and talk about my personal life and I consider her confidential” (Participant 4, girl 17yo, F50)*

Participants reported the following factors as fundamental to establishing a good relationship with their therapist: “She understands me”, “She listens to me” (that is, she puts up with me), “I can speak freely”, “She offers solutions”, and “I feel comfortable”. These are a selection of some of the phrases used by participants who perceive their relationship with their therapist as good.

Typical comments included:

*“I feel an intimacy; she advises how to deal with my difficulties … I feel her close to me and I trust her” (Participant 45, girl 13yo, F34)*

*“My relationship with my therapist is very good … she is very cooperative … she speaks so nicely and clearly …*” *(Participant 18, girl 18yo, bipolar)*

The following was a typical comment illustrating the value of a ‘good’ adolescent-therapist relationship as a facilitator of engagement in therapy. A participant stated,

*“I think that if the therapist succeeds in creating an atmosphere of trust and confidence, then it’d be easier for me to accept the proposed treatment” (Participant 4, girl 17yo, F50)*

#### The (mostly positive) attitudes towards the therapist

The attitudes towards the therapist ranged from mild reservation to strong attachment. Some participants strongly associated their steady commitment to therapy with their good relationship with their therapist. They reported themselves as being strongly attached to their treatment because of their therapist.

*“I don’t think that something would change about the decision I make regarding my relationship with her” (Participant 3, girl 14yo, F39)*

Notwithstanding, a few participants expressed that their attitudes toward the therapist ranged from neutral to mild reservation, which, however, did not amount to actual concerns.

Some participants expressed a wait-and-see attitude towards their therapist. For example, the following comment was typical.

*“I have the judgement to understand if what she proposes to me will help me or not. It’s too early … for the time being I haven’t noticed being affected. I see her positively” (Participant 13, boy 16yo, F40, F51)*

Furthermore, some few participants said they had taken a weak liking to their therapist. Typical comments included:

*“She was nice, nothing special” (Participant 25, boy 13yo, F39; Participant 43, girl 17yo, F32)*

### The adolescents unilaterally determine what constitutes a “good relationship” with the therapist

It is noteworthy that many participants unilaterally set the terms of a “good relationship” with the therapist. Importantly, several participants reported that being allowed to set these terms was a precondition for the continuation of treatment. They appeared to be ready to unilaterally set out the terms and conditions of their therapeutic relationship and terminate the therapy if it opposed their values or their personal beliefs and ideology or if it contradicted basic parts of their character. The need to preserve their selves, instead of a radical change through treatment, seems to be the key point for adolescents. They appeared ready to interrupt treatment unilaterally as an equal partner.

The following comments illustrate this point:

*“… something I can’t accept or it doesn’t fit my personality or character” (Participant 39, girl 15yo, F40 – panic attacks)*

*“I would stop because I was getting frustrated when we disagreed on something” (Participant 25, girl 13yo, F39)*

Surprisingly, while adolescents seek a warm relationship with their therapist, the therapist bringing his or her personal experiences into that relationship may have a tremendous negative impact on adolescents’ treatment engagement. For example, one participant considered this a ‘casus belli’ and expressed a cynical view, alleging that.

*“If she starts bringing her personal stuff in the conversation, that’s a red flag for me” (Participant 14, girl 14yo, F32)*

In addition, it should be noted that some participants used the terms “good relationship” or “bad relationship” [with the therapist], “like [the therapist]”, “I feel good” or “I feel bad” [with the therapist] without providing further clarification of what they meant by these terms. Moreover, adolescents desire good communication with the therapist (Participant 47, girl 16yo, F32) and clarity on what the therapist says (Participant 5, girl 12.5yo, F45, F40).

### The therapy as a means of pursuing and achieving goals to improve the adolescents’ well-being

Psychotherapy emerged in the data analysis as being thought of as a requirement or an effective means of pursuing and achieving adolescents’ goals. From this perspective, setting life goals may be a facilitator for adolescents to engage in their psychotherapy.

#### The goal of eliminating the symptoms and the negative consequences of a mental disorder

The vast majority of the participants in our study seek help to handle their difficulties and get free of symptoms related to their mental disorder, which in all likelihood limit and affect their functional activities of daily living and obstruct the development of social relationships. A total of 22.4% of the participants in our study had sleep disorders. Furthermore, 44.9% of the adolescents in our study were affected by mood disorders, and increased rates of pessimistic symptoms (36.7%), energy reduction (30.6%), and concentration difficulties (28.6%) were observed. It is notable that although the participants in the study stressed the need to develop social relations and be accepted by peers, none, even those who were older, mentioned the creation of romantic relations as a therapy goal. In a related question during their evaluation, they expressed their indifference towards such issues.

##### Focusing on participants’ personal well-being

Some participants were focused on their personal well-being (personal aspect of quality of life) and declared that they pursued the purpose of effectively dealing with their difficulties and challenges. The following were typical comments:

*“I expect to become better and I’m not afraid of anything” (Participant 19, boy 16yo, F42); “… I expect it will relieve me” (Participant 3, girl 14yo, F39); “[ …] … I want to pursue treatment because I will feel better for myself” (Participant 45, girl 13yo, F34).*

Some other participants placed great weight on their symptoms, which, in addition, wreak havoc on their social relationships. A typical comment included:

*“The suggested treatment could help me in various phobias, so as to get over them … if I have issues with my friends, treatment will help me deal with them … I’d like to develop friendships” (Participant 21, boy 17yo, F90)*

##### Focusing on participants’ social well-being

Other participants placed considerable emphasis on their social skills, which were impaired because of their symptoms. Success in achieving the goal of improving social skills and social relationships, especially with peers, emerged as a strong motive for engaging in therapy. For example, participants stated,

*“… I am and make better company with other peers …” (Participant 24, boy 13yo, F90). “… I believe I’ll gain some new friends, it’ll help me live more pleasant moments” (Participant 19, girl 16yo, F42). “I’d like to improve my social relations … I have few friends” (Participant 38, boy 17yo, panic attacks, stress)*

##### The remission of symptoms as a barrier to therapy engagement

Importantly, while the vast majority of participants clearly acknowledged the benefit they received and were willing to undergo treatment, many participants expressed their desire to terminate psychotherapy prematurely once the symptoms subsided or went away. One participant expressed the desire to complete the treatment as quickly as possible. Interestingly, the researchers found that early remission of the symptoms may increase the risk of early withdrawal from therapy. From this perspective, it may be a barrier to therapy engagement. For example, participants stated,

*“I believe I would interrupt treatment if I was reaching a good, very good point … reached the point I wanted” (Participant 13, boy, 16yo, F40, F51); “… if I feel better” (Participant 33, girl, 17yo, F42); “… if I don’t need it anymore” (Participant 3, girl, 14yo, F39).*

#### The goal of personal independence

Some participants regarded therapy as a way to become more independent. For example, participants stated,

*“[ …] I expect to be able to decide for myself and not follow my therapist’s instructions” (Participant 49, boy 13yo, F42)*

*“[ …] I think that … I do this … to be gradually better till I become independent and can find my path alone … to become able to confront the things that scare me alone …” (Participant 4, girl 17yo, F50)*

#### The goal of enhancing self-esteem and developing positive self-image

Some adolescents were motivated to receive treatment out of their need to increase their self-esteem and improve their perception of themselves or their lives.

The following comments illustrate this point:

*“[ …] I become a better person and I believe I’ll make the right choices about my life … I’ll have a better perception of things …” (Participant 9, boy 17yo, F39)*

*“[ …] I’m not afraid of anything, I expect to have less stress and more confidence … a strong character for my life” (Participant 7, girl 14yo, F42)*

#### The goal of becoming able to achieve personal life goals

The goal of becoming able to achieve their plans in personal and professional fields was reported as a factor motivating participants to engage in psychotherapy. Therapy was regarded by participants as a necessary condition to accomplish their personal and professional plans.

Typical comments included:

*“… I think that if I hadn’t chosen to be treated, some future plans would not have been accomplished” (Participant 9, boy 17yo, F39)*

*“My plans for the future were mainly the incentive to accept the proposed treatment” (Participant 4, girl 17yo, F50)*

However, the vast majority of participants did not connect the achievement of future goals with the present treatment, despite the gravity of their current mental health condition.

The majority of adolescents identified their schooling and future career preferences as the main priority. The goals of leaving the family home, moving abroad for school and creating their own family express their deeper need for independence and autonomy.

#### The goal of confessing to a trustworthy person

Three participants regarded the therapy as a confession. They were willing to confess their personal distress to a trustworthy person. For example, participants reflected:

*“… I don’t exactly know [the problem], but speaking helps get it out of you” (Participant 3, girl 14yo, F39)*

*“… I expect to describe what I’ve been through from my father … to say what I have inside of me …” (Participant 41, girl 15yo, F39)*

### The role of peers (ranging from neutral to mildly supportive)

The role of peers ranged from ‘not-so-neutral’ to mildly supportive. While the vast majority of participants had friends and quite a few of them, most participants chose not to announce to their friend group their problem and the fact that they were in treatment. Many participants distinguished between their close trusted friends and their not-so-close friends. Close friends may know about the adolescent’s therapeutic relationship, and they potentially act supportively with emotions of solidarity, understanding and motivation. Most participants mentioned that their attitude towards treatment was not influenced by their friends. Several participants felt support (participant 38, boy 17yo, panic attacks, stress; participant 8, girl 15yo, F50; participant 28, girl 15yo, personality disorder) or were urged towards treatment by friends (participant 14, girl 14yo, F32), and some even stated that they pursued treatment to have a good relationship with their friends.

Typical comments included:

*“If I have problems with my friends, treatment will help me deal with them” (Participant 21, boy 17yo, F90)*

*“… it’s such a case, because the relationship I have with my friends encourages me to try not to be cut off by them again”.* Moreover, the participant clarified “*the relations I have with my friends are generally good, but there are some friends with whom I can talk about personal issues. I trust them more and they encourage me” (Participant 4, girl 17yo, F50)*

However, none of the participants stated that friends played a crucial role in seeking treatment and engaging in it.

### The role of family (ranging from supportive to active)

The role of family ranged from only mildly supportive to strongly supportive. All participants stated they had a good relationship with their family. Very few were slightly cautious in making this statement. The role of the family in the treatment continued to be important, despite the tendency toward independence that characterizes adolescence.

To a greater or lesser extent, family helped the adolescents become willing to undergo treatment. While some adolescents took the initiative to seek therapy, in some other cases, the initiative was taken by family. However, none of the participants reported that they felt forced into psychotherapy. In almost all of the cases, adolescents were backed by family. The adolescents showed a positive attitude towards the supportive role of family.

A few participants clearly highlighted their own leading role in seeking treatment, thus minimizing the supportive role of the family. Typical comments included,

*“I had asked to come” (Participant 14, girl 14yo, F32)*

In some cases, family had the leading role in seeking treatment.

*“Basically, they brought me” (Participant 2, boy 13yo, F51.3)*

Note, however, that several participants declared that family had a supportive role either underpinning the adolescents’ willingness to seek treatment for their mental problems or urging and persuading the adolescents of the value of a treatment. For example, participants stated,

*“In a way yes [my parents brought me here], but I don’t think it’s the main reason” (Participant 8, girl 15yo, F50)*

*“With discussion, they managed to lead me to talk to someone” (Participant 46, girl 13yo, F40)*

Surprisingly, a participant, highlighting the supportive role of family, put it best when she said*, “… when the family doesn’t accept it [the treatment], then the child doesn’t accept it either” (Participant 39, girl 15yo, F40- panic attacks).*

Participants reported as facilitators of their therapy engagement the fact that their therapy might reduce intrafamily conflicts, reduce their aggressive behaviour towards their family and make their family happy. These changes might be beneficial to the adolescents themselves. For example, participants stated,

*“I believe that my decision [to undergo therapy] will improve the bonds between us and I’ll have the support of my parents for anything I want to do” (Participant 3, girl 14yo, F39)*

*“I want my mom to feel good, so I’ll go along with what they ask from me” (Participant 41, girl 15yo, F39)*

### The fear of stigma related to a mental health disorder (as both a barrier and facilitator)

#### The fear of social stigma

The stigma attached to mental illness is very real. Participants fear others knowing that they are in therapy, which may become a barrier to getting therapy. Other participants fear others making fun of them (their mental illness), which may facilitate their obtaining therapy. The fear of stigma related to mental health was both a facilitator and barrier. For example, participants, when talking to the interviewer, reflected:

*“I don’t have a problem because this won’t be known and it will also help me … yeah, since you are not going to say it to anyone, I don’t need to worry …” (Participant 3, girl 14yo, F39)*

*“… if I feel it [therapy] helps me more and I want to finish … I don’t want it much, I want to finish … it helped me already, I don’t want anything more … I’m afraid to stop … some classmates make fun of me” (Participant 10, boy 13yo, F84.5)*

#### Addressing self-stigmatization as a facilitator of therapy engagement

Adolescents’ recognition that they are not dealing with a serious issue seems to function as a relief and encourages them to seek treatment. For example, two participants reflected:

*“Since I know the reason … I am aware of my condition, that it’s not a serious problem or something to worry about, I don’t see it [the treatment] negatively, as another child might, and I am OK with it and myself and I don’t worry” (Participant 13, boy 16yo, F40, F51)*

*“I don’t have a special problem, but I think it would be good to discuss my problems with someone” (Participant 26, boy 13.5yo, F39)*

This may be the reason why adolescents with mental disorders may shift the responsibility for therapy exclusively to parents (see below on the role of family).

Considering all the above-mentioned results and after having ranked the identified barriers and facilitators as major and minor, the findings of this study can be briefly summarized as follows: In the interviews, all of the participants highlighted facilitators of rather than barriers to their treatment engagement. Positive experiences with therapy, namely, positive treatment outcomes and the perceived effectiveness and usefulness of treatment, were reported as strong (“major”) facilitators of therapy engagement for adolescents with mental disorders, whereas negative experiences with therapy (perceived as ineffective and unhelpful**)** were reported as strong (“major”) barriers to it. The participants equally highlighted the importance of eliminating their symptoms and improving their socialization skills. Furthermore, and most importantly, a ‘good’ adolescent-therapist relationship was reported as a strong (“major”) facilitator, whereas negative experiences of participants with their therapist (namely, a “bad” relationship with the therapist) were reported as a strong (“major”) barrier to their therapy engagement. A meaningful adolescent-therapist relationship meant ‘good’ adolescent-therapist interaction, namely, a meaningful, close, trustful, warm, open, communicative and familiar relationship with the therapist in which adolescents feel comfortable. Note, however, that several adolescents unilaterally set the terms of a (perceived as) “good” relationship with therapist. Moreover, goals such as getting rid of the symptoms and negative consequences of the mental disorder, improving personal well-being, and improving social skills and relationships, especially with peers, emerged as strong (“major”) facilitators of therapy engagement. Importantly, early remission of symptoms emerged from the study as a strong (“major”) barrier to therapy engagement for participants. This was described as a factor that can cause a high attrition rate. Among the weaker (“minor”) perceived facilitators were goals such as confessing to a trustworthy person, becoming able to achieve personal expectations and life goals, enhancing independence and self-esteem, and developing a positive self-image. In addition, the (active or supportive) role of family emerged as a facilitator. The stigma related to mental health emerged from the study as both a (“minor”) facilitator of and barrier to therapy engagement for participants. Friends were reported as having a role ranging from neutral to mildly supportive.

## Discussion

The authors of this paper would not expect to find quite different results among adolescents without DMCs, with the exception of results related to the adolescent-therapist relationship. Note, however, that, as the participants in this study were all voluntarily receiving therapy, the authors can imagine that adolescents who were not in therapy by choice might answer differently.

The facilitators of and barriers to psychotherapy engagement that emerged from this study were ranked as major (strong) and minor (less strong) according to their recurrence rate of the related data in the interviews (quantitative criterion) and the emphasis placed on these interview data by participants (qualitative criterion). From this perspective, below, the authors discuss the results of this study. As the authors conducted an inductive content analysis, the structure of this section is based on the key findings of the study.

### The adolescents’ attitudes towards therapy

All participants had a positive attitude towards treatment. The vast majority of the participants in this research clearly acknowledge the benefit of receiving treatment and express explicit desire to undergo treatment. This is not consistent with the findings of prior studies, according to which adolescents frequently do not perceive the need for psychotherapy [4, 54]. It is argued that adolescents may lack the cognitive abilities and experience to fully understand the rationale behind treatment and doubt that it will have any meaningful impact on them [5]. Furthermore, it is argued that adolescents are less able than adults to assimilate and integrate, analyse, synthesize and evaluate the information provided, even though they may completely recognize the (short-term, but not the long-term) benefits of the recommended treatment [19]. Moreover, it should be highlighted that “it is unclear how well adolescents with psychiatric problems appreciate their disorder and treatment recommendations …” [55]. Note, however, that the researchers included only adolescents who we considered competent to make decisions and were already in therapy. As anticipated above in the Introduction section, a subset of adolescents with mental disorders are likely to be decision-making competent in specific contexts. These adolescents can be effectively involved in the SDM process and hence effectively engage in their treatment*.* To that effect, many theorists suggest that “children may have far more potential to understand complex illness concepts than they have previously been given credit for” [56].

Many of the participants were of the opinion that the remission of the symptoms would be a good reason for premature termination of therapy, which in turn may hinder the effective delivery of mental health services [57]. It is important that quite a few adolescents seem to try to “have control” of the therapeutic relationship, setting their own terms for treatment delivery, which they mainly relate to the quality of their relationship with the therapist (see below). This corresponds with adolescents’ desire for immediate results and focus on short-term outcomes and is consistent with the egocentrism of adolescence. Adolescents’ perceived limits to their freedom to “choose”, tendency to regard psychotherapy as an effort to control them and as in conflict with their striving for autonomy, and stereotype-based inaccurate impressions of psychotherapy may be significant barriers to therapy engagement. This is particularly so when adolescents in therapy fail to perceive themselves as needing therapy and participate in therapy because others want them to be in therapy [5]. Αdolescents have a propensity towards risk-taking and short-term reward (reward reactivity) due to asymmetry in the development of various structures in the adolescent brain [23, 27]. In addition, adolescence is characterized by the perception of invincibility and egocentrism (failure to inhibit self-perspective) [58, 59]. Many participants clearly and unequivocally stated that they were afraid of nothing. Naturally, this agrees with the feeling of invincibility and risk-taking during adolescence.

As all of the participants were actively engaged in therapy, it is likely that their prevalent symptoms are potential facilitators of their engagement in therapy. In that regard, the interesting finding that early remission of symptoms may increase the risk of early withdrawal from therapy should be highlighted. A possible explanation may be that the remission of symptoms may considerably reduce both public stigma (related to mental disorder) and self-stigmatization. In addition, with the remission of the symptoms, the need for follow-up is eliminated.

Last, it is noteworthy that our participants did not hold beliefs about mental illness, mental health treatment or terrifying hospital experiences that contributed to poor treatment engagement, as Stafford and Draucker recently found in their study [54].

### The important adolescent-therapist relationship

The vast majority of participants in this study reported that they had developed a meaningful connection with their therapist. They said that this was a significant facilitator of their therapy engagement. They stressed their belief that negative experiences with their therapists would be a major barrier to their therapy engagement. The findings were consistent with previous studies [5, 54]. The literature has long acknowledged the adolescent-therapist relationship as crucial for adolescents’ engagement in treatment [5]. Adolescents’ perceptions of therapists predict therapeutic outcomes [5]. Treatment engagement is decisive in an effective therapeutic process and in achieving successful outcomes and ‘may be particularly relevant early in treatment’ [4]. The better the treatment engagement, the more favourable the therapy outcomes may be.

Several participants mentioned trust in the therapist. Adolescents are more likely to seek health care if their provider guarantees confidentiality, but in providing confidential care, a balance among the needs of the adolescent patient, the parents, and the provider must be considered [60]. In the literature, distrust of psychotherapists is most commonly reported as a barrier to therapy engagement in Latino adult populations. Furthermore, adolescents want a relationship with their therapist that carries a genuine sense of mutual trust [54, 61–68]..

Participants in this study desired a humane, approachable therapist who would collaborate with them and treat them as equals. Therapeutic engagement is “a reciprocal interaction in which both therapist and client(s) have a responsibility for establishing an effective rapport”, namely, creating an optimum working collaboration between the therapist and adolescent [5]. Adolescents should be offered a way to build rapport with the therapist. They should participate fully and engage in positive interactions to achieve successful therapy. Healthcare providers’ ability to engage adolescents increases the likelihood of continuation of treatment [69]. Positive attitudes of psychotherapists towards their patients are more engaging than the more traditional neutral stance often assumed by psychotherapists. Adolescent-therapist agreement, i.e., co-endorsement of aetiological beliefs, may significantly facilitate treatment engagement by promoting communication/openness, adolescent-therapist interaction, and adolescents’ perceived usefulness of treatment [70].

Oetzel and Scherer argue that “succeeding as a psycho-therapist with adolescents can be challenging” [5]. The authors state, “establishing a strong therapeutic alliance with adolescents require that therapists express empathy and genuineness … and increase choice in therapy” [5].

Many participants expressed their need to feel that their therapist would understand them and that he or she would be a source of support. Participants appreciated the fact that the therapist gave them thoughtful and effective advice. This is inconsistent with prior literature [68]. They perceived their therapist as empathic, caring, open, and sincere.

Some participants said, “the therapist understands me”. It is true that both the therapist and adolescent should be minded to ‘understand’. Indeed, reciprocal understanding between the adolescent and therapist is of great importance. Understanding how adolescents perceive mental illness may be important for therapists to improve engagement [71]. If providers motivate adolescents to understand the value of treatment, they may increase the engagement of adolescents, who will be less likely to terminate prematurely [72]. It is crucial to bear in mind that adolescents may undervalue or overestimate the importance of their psychological symptoms and may be ashamed of reporting them [5]. It is possible to identify different treatment engagement profiles [73]. Note, however, that treatment motivation should be distinguished from treatment engagement [74]. Motivational interviewing, used as a pre-treatment intervention, is a promising way to facilitate engagement in adolescent mental health settings [75].

Empathy is necessary for developing a therapeutic alliance with adolescents but is not sufficient. Adolescents appreciate therapists who are committed to them and their well-being.

Some participants said that they wanted the therapist to be precise and that they wanted to feel comfortable with him or her. Sincerity, candour or “being real” with adolescents is a crucial therapy engagement facilitator. Candour implies telling adolescent patients the truth tailored to the adolescents’ developmental capacities. Cognitively immature adolescents require the therapist to use simple inquiries devoid of abstract terms, concrete examples, and guidance on how to establish therapeutic rapport [5].

Some participants stated they were willing to interrupt treatment if it contradicted their values. Respect for the personal values of the adolescent is crucial both in the therapist’s approach and for the adolescent’s therapeutic goals. In relation to the above, it should be mentioned that each minor experiences the outside world in his or her own unique way, even though he or she lives in the same social-cultural context as other children [76].

All the aforementioned features of a therapeutic relationship are necessary to develop an effective SDM process that predicts effective treatment engagement in adolescents. Therapists should establish a climate that enables a thorough exchange with adolescents and their families, which allows for flexible and respectful SDM [77]. Furthermore, therapists should balance the views of parents and children [78] while making every effort to involve the adolescent’s family in the decision-making process [36, 79, 80].

It is important for therapists to go beyond the provision of adequate, clear, concise and unbiased information to the patient [81]. Therapists have to empower and stimulate adolescents to fully engage them in the process of making shared decisions, with their own values derived from their own viewpoint, preferences and emotions [11, 81]. Importantly, the irrational decisions of adolescents that are nevertheless coherent with their “internal rationality” may be regarded as internally reasonable decisions [82]. SDM ‘is increasingly being suggested as an integral part of mental health provision’ [6], especially in the context of child and adolescent psychiatry [7]. However, while a subset of adolescents are decision-making competent [17, 83], therapists may have challenges engaging adolescents with mental disorders in SDM [6, 84].

### The therapy as a means of achieving goals to improve adolescents’ well-being

Most participants did not correlate their goals for the future with their present treatment. The fact that adolescents feel “invincible” and have a short-term perspective probably helps them deal with weaknesses. However, for some, the realization of their aspirations and expectations constituted a facilitator of their treatment engagement. In that sense, it is important to note Almroth et al.’s statement that “interventions aimed at increasing aspirations and engagement in school may prevent mental health problems in adolescence” [85]. Furthermore, leaving home and university studies seem to be important aspirations for adolescents in the research and an element of autonomy and independence from family safety and protection. The selection of a foreign country for further schooling appears to be an idealistic choice that signifies social recognition and financial independence.

### The role of peers

Acceptance by peers and improvement of social skills as essential preconditions for acceptance in the social environment seem to constitute a strong motive for seeking treatment. This is not surprising. Adolescents pursue their need for independence by placing a considerable emphasis on attempting to shift from dependency on parents and family towards greater belonging among peers [5]. Many writers highlight the participation of adolescents in groups of peers as a necessary process of maturation, experimentation and finally discovery of the true self [86–88].

Potential social stigma against mental illness and their inability to deal independently with their difficulties pushes adolescents to conceal their receipt of treatment from their social circle while they simultaneously seek help to integrate into it and to develop trusting relationships with peers. The mental disorder-related stigma attitudes of peer groups towards adolescents in psychotherapy may result in adolescents feeling scorned [5].

However, our research has shown that stable and tested friend relationships can function to encourage adolescents towards therapy and that there is a distinction between “companions” and “friends”. In that regard, it should be highlighted that peers, even though they often are not part of the medical conversation, may actually motivate a (mentally or not) ill adolescent to be more socially active, thus improving his or her DMC [23]. Indeed, in a peer context, an observed adolescent may want to send a social signal to his or her peers [29]. The developmental processes that underlie the sensitivity of adolescents to peer influence are poorly understood [29]. At any rate, it should be highlighted that the influence of peers on outcomes in psychiatric mental health contexts remains poorly understood [19].

### The role of family

In almost all of the cases, adolescents were backed by family to some extent. Adolescents in the study acknowledged the important role of family in their decisions. Tsiantis et al. state that when an adolescent comes for therapy, he or she has already been exposed to familial, friend and social influences, and this can make his or her attitude towards treatment positive [89].

It is very important that parents first note there is a problem and persuade the adolescent to seek treatment, because, as a participant said, “if the family doesn’t accept the problem, then nor does the child” [89]*.* Although this was not the case for the participants in our study, adolescents often underestimate the importance of and need for treatment and are involuntarily referred by parents and other health care providers [90]. Furthermore, not only in mental health care but also in other health care contexts, parents may facilitate adolescents’ DMC more than physicians do, creating the context for adolescents’ competent decision making [6]. Parents can be a barrier to or facilitator of an adolescent’s treatment decision [91]. The American Academy of Pediatrics claims that parents have no absolute legal right to make autonomous treatment decisions regarding their children [17]. Parents do have a responsibility to preserve family relationships and further the best interests of their children. According to the model of constrained parental autonomy, parents can “balance the “best interest” of the minor patient with his or her understanding of the family’s best interests …” [17]. At any rate, it is important to highlight the fact that parents and physicians do not always understand what is in adolescents’ best interest [92]. When parents perceive their children’s mental health problems to be serious, they are more likely to seek mental health therapy for their children [54, 93]. However, while the majority of families perceive the need for treatment, that perceived need may not be associated with treatment engagement [94].

Adolescents’ and parents’ needs and perceptions regarding the need for and barriers to care may differ. It is important to align adolescents’ and parents’ needs throughout treatment [91]. Moreover, a collaborative relationship between the family and the health provider may increase engagement [4].

Additionally, it is important to bear in mind that the influences of parents and family on outcomes in psychiatric mental health contexts are poorly understood [19].

### The fear of stigma related to mental health disorder

The fear of mental health-related stigma emerged in the interview excerpts regarding the interviewee-therapist and interviewee-peer relationships. The role of the stigma that many adolescents associate with psychotherapy should be noted. In the literature, stigma associated with mental illness is most commonly reported as a potential but fundamental attitudinal barrier to seeking and engaging in mental health treatment among adolescents with mental disorders [54, 62, 66–69, 95, 96].

Unlike in previous research [54], the participants in this study did not report bad patient experiences in the hospital. Financial or infrastructural barriers that prevent access to care were not identified as affecting adolescents’ treatment engagement or health-seeking behaviours with regard to mental health care.

### Limitations

The authors of this paper did not explore the perceptions of the participants’ families. Furthermore, they did not explore how developmental factors may help or hinder therapeutic engagement. Moreover, as in this study the authors combined participants from across a broad spectrum of diagnosis areas and some of the participants could not be classified in mutually exclusive diagnosis areas, they did not conduct different data analyses for each diagnosis-related group. In addition, it should be noted that the participants in this study were consensually engaged in ongoing treatment for at least four months at the time of the interview. Last, the researchers tried their utmost to better assess the participants’ DMC. However, this assessment involved a degree of uncertainty and therefore may be seen as a limitation.

### Ιmplications

For the most part, the findings enhance prior studies. However, the authors of this paper identified some nuances that can be used to inform the development of interventions that might contribute to enhancing facilitation techniques and reducing barriers to mental health care engagement among adolescents. For instance, with the remission of symptoms, adolescents are likely to terminate treatment prematurely. To address this issue, therapists should highlight the long-term benefits of therapy. Moreover, therapists should highlight the association between these long-term benefits and the achievement of adolescent clients’ goals and encourage adolescent clients to consider the prospect of achieving enhanced family well-being because of the improvement in their mental health. Being a psychotherapist for adolescents is challenging and is a role that needs to be supported.

## Conclusions

A number of more or less strong barriers and facilitators were identified. Positive experiences with therapy were reported as strong (“major”) facilitators of therapy engagement for adolescents with mental disorders, whereas negative experiences with therapy were reported as strong barriers to it. Furthermore, and most importantly, a “good” adolescent-therapist relationship was reported as a strong facilitator, whereas negative experiences of participants with their therapist were reported as a strong barrier. Moreover, goals such as getting rid of symptoms, improving personal well-being, and improving social skills and relationships (especially with peers) emerged as strong facilitators of therapy engagement. Importantly, the early remission of symptoms emerged in the study as a strong barrier to therapy engagement for participants. Among the weaker (“minor”) perceived facilitators were goals such as confessing to a trustworthy person, becoming able to achieve personal expectations and life goals, enhancing independence and self-esteem, and developing a positive self-image. The (active or supportive) role of family emerged as a facilitator. The stigma related to mental health emerged as both a (“minor”) facilitator of and barrier to therapy engagement for participants. Friends were reported as having a role ranging from neutral to mildly supportive. Adolescents’ interactions with their social environment might be a facilitator of or a barrier to their treatment engagement.

For the most part, the findings of this study are consistent with the findings of previous research on the topic of interest. They enhance the findings of prior studies. It is noteworthy that the assessed-as-decision-making competent participants in this study had a (mostly strong) positive attitude towards their treatment engagement. Furthermore, the authors of this study identified some nuances that, to some extent, extend the findings of previous studies and might be used by therapists to enhance adolescents’ treatment engagement. For instance, considering that symptom remission is likely to cause adolescents to terminate the treatment prematurely, highlighting the achievement of adolescents’ future goals as well as the prospect of achieving enhanced family well-being because of the improvement in adolescents’ mental health may contribute to reducing the attrition rate. This implication for therapists can be used to guide them and lead them to assume more responsibility and initiative for enhancing treatment engagement in adolescents with mental disorders.

## Supplementary Information

**Additional file 1.**

## Data Availability

The datasets used and analysed in this study are available from the corresponding author on reasonable request.

## Abbreviations

DMC: Decision Making Competence/Capacity
SDM: Shared Decision Making
