# Supplementary material for: Perceived facilitators of and barriers to mental health treatment engagement among decision-making competent adolescents in Greece
Source: BMC Psychiatry. 2021 Sep 22;21:461. doi: 10.1186/s12888-021-03471-0 (PMC8456699; doi:10.1186/s12888-021-03471-0)
Supplement: Supplementary file 1 — Additional file 1. [file 12888_2021_3471_MOESM1_ESM.docx]

**Additional File 1**

**(Additional quotations)**

1. **The adolescents’ attitudes towards therapy**
   1. **Strong commitment to therapy**

*“…if, knock wood, I had something and was in hospital for days, if someone was injured, then I believe I’d interrupt…” (Participant 49, boy 13yo, F42)*

*“I’m willing to pursue treatment; I believe I’m not afraid of anything and it only has benefits to offer” (Participant 9, boy 17yo, F39)*

- 1. **Ineffectiveness of therapy as a barrier**

*“I’d interrupt it, if I saw it didn’t help me” (Participant 12, boy 15yo, socialization problems)*

*“I have applied what she [the therapist] proposes to me and it leads to good results and they help me personally, so whatever she says, I listen” (Participant 8, girl 15yo, F50)*

*“As I have mentioned, it [the treatment] influences me only positively” (Participant 9, 17yo, F39)*

*“…I take it cool… in the beginning I didn’t expect much, now I hope for the best” (Participant 28, girl 15yo, personality disorder)*

*“I intend to stop it, if it doesn’t help me” (Participant 43, girl 17yo, F32)*

*“…if everything continues to be so bad or if it makes me feel more numb or empty” (Participant 30, girl 13yo, F32)*

*“…if I felt it doesn’t help me” (Participant 28, girl 15yo, personality disorder)*

*“…if I saw it is ineffective” (Participant 19, girl 16yo, F42)*

*“…some kind of health problem it could cause me, what the therapist recommends” (Participant 24, boy 13yo, F90)*

1. **The crucial role of the adolescent-therapist relationship**
   1. **The (perceived as) ‘good’ adolescent-therapist relationship**

*“Very good and friendly” (Participant 24, boy 13yo, F90)*

*“My relationship with my therapist plays a major role in my decision to accept the proposed treatment” (Participant 21, boy 17yo, F90)*

*“I trust her” (Participant13, boy 16yo, F40, F51)*

*“She’s very good, I solve my problems” (Participant 10, boy 13yo, F84.5)*

*“… I love her a lot” (Participant 17, boy 14yo, F84.1)*

*“My relationship with the therapist is very good; he makes me feel close to him” (Participant 12, boy 15yo, socialization problems)*

*“I trust him” (Participant 2, boy 13yo, F51.3)*

*“She is very good, I have worked with her for quite some time. I trust her” (Participant 33, girl 17yo, F42)*

*“I can say she’s like a friend of mine” (Participant 39, girl 15yo, F40- panic attacks)*

*“I have a good relationship” (Participant 49, boy 13yo, F42)*

*“…friendly” (Participant 34, girl 13yo, academic difficulties)*

*“I like her, she seems nice” (Participant 41, girl 15yo, F39)*

*“The relationship with my therapist is good” (Participant 30, girl 13yo, F32)*

*“We have a good relationship… I like her very much” (Participant 3, girl 14yo, F39)*

*“[The relationship with my therapist is] very good; I can open up to my doctor, and we also have fun, and it’s very nice… I also trust her” (Participant 8, girl 15yo, F50)*

*“She understands me” (Participant 25, girl 13yo, F39)*

*“[Our relationship is] very good…she understands my character and who I am as a person” (Participant 9, boy 17yo, F39)*

*“My relationship with my therapist is pretty good… she helps me solve my problems… I feel comfortable…” (Participant 7, girl 14yo, F42)*

*“There is intimacy, but mainly I can trust her and I rely on the things she says, ‘cause I believe she speaks objectively… there is trust” (Participant 19, girl 16yo, F42)*

*“She listens to me and understands me, something I greatly appreciate” (Participant 3, girl 14yo, F39)*

*“My relationship with my doctor is very good… and she understands me” (Participant 31, girl 16yo, F50)*

*“My relationship with the therapist is pretty good, as we talk about my relationships with other people” (Participant 21, boy 17yo, F90)*

- 1. **The (mostly positive) attitudes towards the therapist**

*“I don’t think I could interrupt my treatment without my therapist saying so” (Participant 18, girl 18yo, bipolar)*

However, some participants stated,

*“**It’s too early to say we have a special relationship” (Participant 28, girl 15yo, personality disorder)*

*“Neutral relationship. I don’t know her well yet” (Participant 9, boy 17yo, F39)*

*“…nice, I don’t dislike her” (Participant 5, girl 12,5yo, F45, F40)*

*“(hesitates to answer) yeah, she’s formal but not to the point of becoming indifferent, that’s that” (Participant 47, girl 16yo, F32)*

*“I don’t know her yet. She seems nice” (Participant 29, boy 16yo, F39)*

- 1. **The adolescents unilaterally determine what constitutes a “good relationship” with the therapist**

*“If I didn’t like him and we disagreed about this treatment, I’d definitely interrupt it” (Participant 7, girl 14yo, F42)*

*“…if it is clear what she says, then yes”* *(Participant 5, girl 12,5yo, F45, F40)*

*“If I had a different doctor where we wouldn’t have such communication and a good relationship, it’d be more difficult for me to apply some things” (Participant 47, girl 16yo, F32)*

*“It depends on how I feel” (Participant 33, girl 17yo, F42)*

*“[I’d interrupt] if I felt bad, but I don’t think something like that would happen” (Participant 26, boy 13,5yo, F39)*

*“If it wasn’t good [the relationship with the therapist], I wouldn’t follow it [treatment]” (Participant 43, girl 17yo, F32)*

*“I believe, yeah, if I didn’t like her as much as I do, I may have been more hesitant in following the treatment” (Participant 4, girl 17yo, F50)*

*“A bad relationship with the therapist [is a reason for interrupting treatment]” (Participant 45, girl 13yo, F34)*

*“It would make me interrupt it [treatment]… when the doctor would make me feel uncomfortable” (Participant 12, boy 15yo, socialization problems)*

1. **The therapy as a means of** **pursuing and achieving goals to improve adolescents’ well-being**
   1. **The goal of eliminating the symptoms and the negative consequences of a mental disorder**
      1. **Focusing on participants’ personal well-being**

*“I believe it will help me… feel better” (Participant 43, girl 17yo, F32)*

*“I think that I have someone I will talk to about my problems and he will really help me… I’m not afraid of something regarding treatment; on the contrary, I feel I can be greatly helped… I expect that … I’ll be able to clarify some of my doubts… perhaps be able to deal better with the difficult situations in my life” (Participant 3, girl 14yo, F39)*

*“…it helps me…yeah…to deal with my difficulties… it feels difficult ‘cause we are talking about my difficulties…” (Participant 10, boy 13yo, F84.5)*

*“I came for the scribbles in my writings… I’d like to see what causes my scribbles… I’m not afraid of anything [because of the treatment]” (Participant 9, boy 17yo, F39)*

*“I think I have someone next to me and I’m sure they will help me in everything that’s happening… I’m willing to pursue treatment because I believe it will help me and I’ll be fine… it’ll help me control my emotions” (Participant 18, girl 18yo, bipolar)*

*“…yes, since it helps me… cause I have these panic attacks, I believe I’ll be helped” (Participant 39, girl 15yo, F40 – panic attacks)*

*“I have a problem with my sleep and a little more with my appetite… [I don’t want to] feel numb or empty” (Participant 30, girl 13yo, F32)*

*“…it will help me… not wake up at night” (Participant 2, boy 13yo, F51.3)*

*“… it helps me… some things can be fixed… [I’ll stop treatment] when the phobias stop” (Participant 46, girl 13yo, F40)*

*“I think it will help me eliminate stress and the thoughts that overwhelm me… but I’m afraid it’ll be quite difficult to follow some of the steps… by freely expressing my questions and thoughts to a specialist who will give me suitable advice… I consider mental calmness and maintaining good relations with the social environment very important…” (Participant 4, girl 17yo, F50)*

*“[I believe treatment] will help me approach people more easily… yeah, I’m willing [to receive treatment]…to feel better” (Participant 29, boy 16yo, F39)*

*“…I don’t feel something, I feel it’s something that helps me… yes (with certainty)… I expect to become a more normal child than I am and become better acquainted with my peers (Participant 24, boy 13yo, F90)*

*“Yes, I’m willing to receive treatment. I know it will solve the problem I have with myself and others to a point and then I’ll feel better with myself… my plans for the future are to become more social talking to many people, even from other countries…” (Participant 12, boy 15yo, socialization problems)*

*“I find it normal [being in treatment] because I’m aware of my condition… now yes, I hope it’ll help me in my daily mood and how I handle situations… I expect it to improve my mood and along with my own efforts to improve some parts of my life… in the social part I believe I’ll gain some new friends, it’ll help me live more pleasant moments… if I didn’t accept treatment, my initial solution would be suicide” (Participant 19, girl 16yo, F42)*

*“I feel relief and I have guidance on how to elaborate on emotions and situations… I feel safe and at ease to express my thoughts and emotions…I’d like to improve my social relations… I have few friends” (Participant 38, boy 17yo, panic attacks, stress)*

*“[I want to] be able to develop friendships with other children… to start a conversation, to be myself, because many times I’m afraid to speak” (Participant 35, boy 17yo, F84.1)*

*“[I want to] solve some problems I had with my classmates at school” (Participant 25, girl 13yo, F39)*

- - 1. **Focusing on participants’ social well-being**
    2. **The remission of symptoms as a barrier to therapy engagement**
  1. **The goal of personal independence**
  2. **The goal of enhancing self-esteem and developing positive self-image.**

*“[I believe treatment] is an experience that will quite help me… I’m available and pretty optimistic about the result… I expect to be helped in the issue I’m having and also how I see myself” (Participant 31, girl 16yo, F50)*

*“[…] I’m not afraid, I want to see how I am inside… to learn how I am… I want to look at myself, what I’ve become” (Participant 16, boy 13yo, F32)*

*“[…] it makes me think more rationally and I deal better with the issue I’m having… as I am better with myself, I will also be better with others” (Participant 13, boy 16yo, F40, F51)*

- 1. **The goal of** **becoming able to achieve personal life goals**

*“I hope to enter the school of my preference, and then I don’t know what will happen… I want to leave home” (Participant 47, girl 16yo, F32)*

*“… to study psychiatry and go to America” (Participant 8, girl 15yo, F50)*

*“…I hope to succeed in the university entrance exams” (Participant 39, girl 15yo, F40- panic attacks)*

*“I’d like to study and feel better” (Participant 33, girl 17yo, F42)*

*“… study here and leave for Germany, study there as well” (Participant 4, girl 17yo, F50)*

*“I believe that [the treatment] will help me achieve some goals in my life” (Participant 12, boy 15yo, socialization problems)*

- 1. **The goal of confessing to a trustworthy person**

*“I think it’s something good, ‘cause there were periods previously in which I was much worse, it’s something that helped me, I believe it’s good… I’m being helped initially because I express myself to someone, I say what I feel… those things I’m afraid to say to my friends and my parents” (Participant 47, girl 16yo, F32)*

1. **The role of peers (ranging from neutral to mildly supportive)**
2. **The role of family (ranging from supportive to active)**

*“It was more my decision” (Participant 25, girl 13yo, F39)*

*“They’re quite cooperative in the treatment I’m having, and they urge me on” (Participant 4, girl 17yo, F50)*

*“They’ll influence me positively because I believe they’ll support me and they’d surely suggest it themselves” (Participant 12, boy 15yo, socialization issues)*

*“…when the family doesn’t accept it [the treatment], then the child doesn’t accept it either” (Participant 39, girl 15yo, F40- panic attacks)*

*“…no, my mom told me to come after she assured me that there’s no problem and I also believe that there’s no problem” (Participant 26, boy 13,5yo, F39)*

*“My relationship with my mom, that persuaded me” (Participant 10, boy 13yo, F84.5)*

*“My relationship with my family contributes to my decision to undergo the proposed treatment because if something that happens in my family bothers me, treatment will help me to not be bothered anymore.” (Participant 21, boy 17yo, F90)*

*“First, I believe that tensions, especially with my mom, will diminish. My dad is already more understanding” (Participant 19, girl 16yo, F42)*

*“It affects me because I understand they also find it difficult with my condition” (Participant 33, girl 17yo, F42)*

*“Since I understand what is good for me, it’ll also be good for my parents ‘cause they’ll watch me get better” (Participant 13, boy 16yo, F40, F51)*

1. **The fear of stigma related to mental health disorder (as both a barrier and facilitator)**
   1. **The fear of social stigma**
   2. **Addressing self-stigmatization as a facilitator of therapy engagement**
